# Association between BMI trajectories in late-middle age and subsequent dementia risk in older age: a 26-year population-based cohort study

**DOI:** 10.1186/s12877-023-04483-z

**Published:** 2023-11-24

**Authors:** Zijian Qin, Zheran Liu, Ruidan Li, Yaxin Luo, Zhigong Wei, Ling He, Yiyan Pei, Yonglin Su, Xiaolin Hu, Xingchen Peng

**Affiliations:** 1 Department of Biotherapy and National Clinical Research Center for Geriatrics, Cancer Center, West China Hospital, Sichuan University, Chengdu, 610041 Sichuan China; 2 Department of Epidemiology and Biostatistics, West China School of Public Health and West China Fourth Hospital, Sichuan University, Chengdu, 610041 Sichuan China; 3 West China Hospital, Sichuan University, Chengdu, 610041 Sichuan China; 4 West China School of Nursing, West China Hospital, Sichuan University, Chengdu, 610041 Sichuan China

**Keywords:** Dementia, Body mass index, Population-based cohort study, Genetic performance

## Abstract

**Background:**

The association between body mass index (BMI) and dementia risk differs depending on follow-up time and age at BMI measurement. The relationship between BMI trajectories in late-middle age (50–65 years old) and the risk of dementia in older age (> 65 years old) has not been revealed.

**Methods:**

In the present study, participants from the Health and Retirement Study were included. BMI trajectories were constructed by combining BMI trend and variation information. The association between BMI trajectories at the age of 50–65 years and dementia risk after the age of 65 years was investigated. Participants with European ancestry and information on polygenic scores for cognitive performance were pooled to examine whether genetic predisposition could modify the association.

**Results:**

A total of 10,847 participants were included in the main analyses. A declining BMI trend and high variation in late-middle age were associated with the highest subsequent dementia risk in older age compared with an ascending BMI trend and low variation (RR = 1.76, 95% CI = 1.45–2.13). Specifically, in stratified analyses on BMI trajectories and dementia risk based on each individual's mean BMI, the strongest association between a declining BMI trend with high variation and elevated dementia risk was observed in normal BMI group (RR = 2.66, 95% CI = 1.72–4.1). Similar associations were found when participants were stratified by their genetic performance for cognition function without interaction.

**Conclusions:**

A declining BMI trend and high variation in late-middle age were associated with a higher risk of dementia. Early monitoring of these individuals is needed to prevent dementia in older individuals.

**Supplementary Information:**

The online version contains supplementary material available at 10.1186/s12877-023-04483-z.

## Background

Dementia is a condition involving a group of symptoms causing serious cognitive impairment, which results in a loss of autonomy [1]. As dementia is the major cause of disability in elderly people, it is responsible for the serious burden of disease and economic cost [2]. With the global population aging rapidly in the last few decades, over 55 million people are living with dementia, and the number could reach 78 million by 2030 [1, 3]. Although drugs (e.g., lecanemab) have been developed to modify the pathological process of dementia, there is currently no drug that can cure dementia [4, 5]. Thus, the identification of modifiable risk factors to effectively prevent or delay dementia is critical and urgent.

Several studies verified that abnormal body mass index (BMI) was associated with many diseases such as Parkinson’s disease, cardiovascular diseases, and respiratory diseases [6–9]. However, BMI at different ages seemed to have different, even opposite effects on dementia risk, which was quite distinct from other diseases [10, 11]. High BMI in middle age increased the dementia risk [12, 13], whereas overweight and obesity in later life were associated with a lower dementia risk [14]. One previous study also described the association of higher BMI with dementia varies depending on the follow-up time [15]. With short follow-up, high BMI was associated with increased risk of dementia while the association reversed with longer follow-up [15]. Besides the effects of absolute BMI on dementia risk, a few studies focus on the association of relative weight changes and dementia [16, 17]. Preliminary evidence suggested large weight loss in the elderly was associated with increased dementia risk [16] and stable BMI was associated with the lowest dementia risk [17].

Late-middle age is the period that bridges the middle age and older age, in which health problems are linked to cumulative effects of emerging risk factors [18–20]. Little evidence has been provided about BMI changes in late-middle age and subsequent dementia risk in older age, especially considering fluctuation in BMI and the inherent genetic predisposition regarding general cognitive function. The present study constructed BMI trajectories as a new indicator to consider the BMI trend and variation at the same time. Among participants with European ancestry, we also evaluated whether genetic predisposition regarding general cognitive function could modify this association.

## Method

### Population selection and cohort design

The study was designed based on the HRS, a large national panel survey that collected information from approximately 20000 individuals over the age of 50 years and their spouses in the United States once every two years from 1992 to 2018. The details of the HRS’s cohort design and surveyed information were described previously [21]. In general, in each wave, individuals were asked to answer preset questions mainly about their health, psychosocial and economic circumstances. After 2006, additional data including data on biomarkers and genetics were also collected from individuals. The HRS was supported by the National Institute on Aging (NIA U01AG009740) and the Social Security Administration and was conducted by the University of Michigan.

In our study, we included participants aged 50–65 years available BMI data for at least two waves and calculated cognitive performance scores larger than 6. Participants were excluded if they had (1) a mean BMI less than 18.5 kg/m^2^, considering that underweight BMI might be a preclinical manifestation of dementia; (2) extreme BMI values (bottom 0.5% and top 0.5%); (3) a calculated cognitive performance score below 6 at 50–65 years of age; or (4) unavailable data on cognitive performance after the age of 65 years. A detailed flow chart with the exact number of participants included or excluded at each step is presented in Figure S1.

### Measurements of exposures and outcomes

The period of late-middle age was defined as 50–65 years of age while older age was defined as after 65 years of age. The age of each individual at each wave was calculated as the date of the interview minus the date of birth. A self-reported (or proxy-reported) BMI was acquired at each wave. It was calculated using the self-reported weight divided by the square height. BMI trajectories were constructed by combining the BMI trends (the slope of a mixed-effect model: ascending BMI and descending BMI) and variations (the coefficient of variation of BMI: high variation and low variation) for each individual. The definitions of BMI trend and variation were as follows:

The details of extracting BMI trend for each individual were reported in our previous study [22]. In general, we employed BMI data collected repeatedly between the ages of 50 and 65 years for each participant to build a multilevel mixed-effect model. This model was utilized to estimate the relationship between BMI and the age at which the BMI measurement was taken. In this analysis, the dependent variable was the series of BMI measurements, while both the intercept and the age at the time of measurement were considered as random effects at the individual level. The model coefficient to the age at which each BMI measurement was recorded, referred to as the "slope," was extracted to represent the BMI trend. Specifically, each one unit increase in the BMI trend represented one BMI increase per year in one individual in late-middle life and vice versa. The BMI trend was further classified into BMI and declining BMI groups according to whether the value of the BMI trend was larger than 0 or less than 0. Also, in additional analysis, we refined the categorization of BMI trends into three distinct classes, classified by the distribution of BMI trend across the dataset. We identified "BMI ascend" for trends situated in the top 40% (BMI trend > 0.072), "BMI stable" for trends in the middle 40–60% range, and "BMI decline" for trends found within the lower 40% (BMI trend < 0.0049).

BMI variation were calculated using the coefficient of variation of BMI (the ratio of the standard deviation of BMI to the average BMI of the observed repeated BMI measures in at 50–65 years of age) for each individual. Then participants were divided into high variation group and low variation group according to the mean BMI variation (lower group: BMI variation < 4.886; higher group: BMI variation ≥ 4.886).

The method of defining dementia in HRS cohort was developed by Langa and Weir [23]. Generally, they developed an approach to evaluate dementia and cognitive impairment without dementia that would produce the same population distribution of cognitive states assessed by the Aging, Demographics, and Memory Study (ADAMS) [24], which is a subcohort from the HRS that underwent formal evaluation of clinical dementia ratings. The calculation of cognitive score was based on the test results of the participants’ immediate and delayed recall items, the serial 7 s test, and backward counting. In each wave, participants would receive these cognitive tests. The total cognition score ranging from 0–27 and participants with scores ranged from 0–6 were defined as demented. The Langa–Weir approach was assessed and validated by Crimmins et al. and showed good comparability with the neuropsychological dementia results [25].

### Covariates

Common sociodemographic characteristics, lifestyle factors, and the history of medical conditions were selected as covariates. The covariates included sex (male and female), race (white, black/African American and other), marital status (married, never married, separated/divorced and widowed), Year at aged 65 (1990–2000, 2001–2010, and 2011–2020), education levels (higher, referred to participants with 13 or more years of education; lower, referred to participants with less than 13 years of education), smoking status (current, ever, never), alcohol consumption status (Ever drinks alcoholic beverages: 'Yes' refers to individuals who have reported ever having a drink of alcohol, and 'No' refers to those who have never consumed any alcohol."), and history of medical conditions (i.e., hypertension, heart disease, stroke, arthritis, diabetes, and lung disease, determined through participant interviews using yes or no questions.). Covariates were incorporated into the generalized linear model to account for potential confounding factors.

### Polygenic score (PGS) for general cognitive function

In the HRS, the PGS of general cognitive function was calculated using the results from Davies et al.’s work based on the cohorts for Heart and Aging Research in Genomic Epidemiology, the Cognitive Genomics Consortium consortia, and the UK Biobank [26]. In general, the cognitive and genetic data of 300,486 individuals aged 16–102 years with European ancestry were pooled to perform GWAS meta-analyses of the general cognition function. To calculate the PGSs for HRS respondents, SNPs weights from a GWAS with several National Heart, Lung, and Blood Institute cohorts, and the HRS removed from the meta-analysis. Eventually, the PGS of 12,090 individuals in HRS were calculated with standardized within ethnicity to a standard normal curve (mean = 0, standard deviation = 1). Principal component (PC) analysis was performed to provide sample eigenvectors and was used as a covariate for adjusting the possible population stratification. Details on PGS construction in the HRS cohort were reported on their official website (https://hrs.isr.umich.edu/news/data-announcements/hrs-polygenic-scores-2006-2010-genetic-data).

### Statistical analyses

Restricted cubic spline analysis with 3 knots was conducted based on a logistic regression model to test the nonlinear association between BMI variation and BMI trend. A generalized linear model (with a log link and Poisson distribution) was conducted to investigate the association of the mean BMI, BMI variations, and trajectories in late-middle age with dementia events in older age. Adjusted risk ratios (ARRs) and 95% confidence intervals (95% CIs) were calculated to estimate the effect. Four models were constructed. Model 1 was the univariate model. Model 2 was additionally adjusted for unmodifiable sociodemographic variables including sex, ethnicity, education level, marital status, and year at aged 65. Model 3 was additionally adjusted for potential modifiable variables including smoking status, alcohol consumption, and history of medical conditions. In Model 4, we introduced an additional adjustment by incorporating participants' mean BMI during the ages of 50 to 65 years. This adjustment aimed to account for the potential confounding effect of BMI itself on the subsequent dementia risk. In each model, participants with any missing data were excluded from the model construction. Moreover, we examined whether there was an association with different periods, and no significant interactions were found (*P* = 0.085 for BMI trend and *P* = 0.324 for BMI variation).

We conducted specific stratified analyses to investigate the potential impact of BMI trajectories on dementia risk in conjunction with genetic predisposition to general cognitive function. Initially, we assessed the influence of Polygenic Score (PGS) quartiles, where the top and bottom quartiles represented high and low PGS, respectively, on future dementia risk. This step aimed to validate the relationship between PGS for general cognitive function and actual dementia risk in older adults. Subsequently, we estimated the ARRs of BMI trajectories on the risk of subsequent dementia, after accounting for the influence of PGS. This adjustment allowed us to mitigate the potential confounding effect arising from participants' genetic predisposition to general cognitive function. Lastly, we calculated ARRs while stratifying for PGS and explored potential interactions. This analysis aimed to discern whether the association between BMI trajectories and subsequent dementia risk varied among different genetic predisposition groups.

Subgroup analyses were conducted to examine the modification effects of exposure on sex, race, educational, marital status, education level, smoking status, alcohol consumption status, and history of medical conditions. Several sensitivity analyses were conducted to test the robustness of the results from the main analyses. First, we reconducted our analyses by including participants who had at least three BMI records in late-middle age. Second, we used the exact changes in BMI between the last record and the first record instead of using the BMI trend derived from the multilevel model to test the robustness of the BMI trend. Third, a Cox proportional hazard model was constructed to further examine the association of BMI variations and trends with dementia. The endpoint was the first occurrence of dementia. The follow-up time was defined as the period from the age of 65 years to the age at the interview in which dementia was first occurred or the latest interview with available information, whichever came first. Finally, given the association between missingness and the covariates and BMI trajectories, we employed multiple imputation on our entire cohort (*n* = 22,972) to validate the estimates produced from the complete case analyses in our main analyses [27]. Multiple imputation involves utilizing different techniques to impute missing values. Specifically, predictive mean matching is employed for numerical data, logistic regression imputation is used for binary data, polytomous regression imputation is applied for unordered categorical data with more than two levels, and the proportional odds model is utilized for ordered categorical data with more than two levels.

All analyses were conducted using R 4.0.2. We utilized the lmer function from the R package lme4 to construct the mixed-effect models, the glm function from the R package stats to build the generalized linear model, the mice function from the R package mice to perform multiply imputation and the rms R package to conduct restricted cubic spline analysis. A two-sided *p* < 0.05 was considered statistically significant.

## Results

### Participant characteristics

A total of 10,847 participants were included according to the inclusion and exclusion criteria. With a 7.91 year median follow-up after the age of 65 years, 871 dementia events were identified. During the period when participants were between the ages of 50 and 65 years, the average number of BMI measurements in which they participated was 5.23, with a standard deviation of 1.63. The average follow-up time for participants' BMI was 8.62 years, with a standard deviation of 3.31 years.

When comparing the characteristics of participants who were included in the study to those who were excluded due to a lack of cognitive follow-up information, several differences were observed. Excluded participants were more likely to be male, have higher levels of education, be unpartnered, consume alcohol, have fewer documented medical conditions, and exhibit obesity during the age range of 50–65 years (Table S1).

As shown in Table 1, 3203, 4436, and 3208 participants were classified into a normal, overweight, and obese mean BMI groups, respectively. Among the participants, those in the obese group showed high BMI variation and most of them experienced BMI increases in late-middle age. Moreover, obese individuals were more likely to be black or African American, widowed or separated/divorced, nonsmokers or alcohol consumers with lower educational levels. BMI trend, BMI variations and average BMIs at 50–65 years of age were correlated with each other (Figure S2). Additionally, the baseline characteristics of BMI indicators, categorized based on cognitive status after the age of 65 were shown in Table S2.

**Table 1:** The characteristics of the included participant at their age 65, stratified by the mean BMI between ages 50 and 65

	**Normal**	**Overweight**	**Obese**	**Overall**
	**(*N* = 3203)**	**(*N* = 4436)**	**(*N* = 3208)**	**(*N* = 10847)**
**BMI trend at age 50–65**				
Decline	1685 (52.6%)	1625 (36.6%)	844 (26.3%)	4154 (38.3%)
Ascend	1518 (47.4%)	2811 (63.4%)	2364 (73.7%)	6693 (61.7%)
**BMI variation at age 50–65**				
Mean (SD)	0.0413 (0.0304)	0.0472 (0.0343)	0.0587 (0.0424)	0.0489 (0.0365)
Median [Min, Max]	0.0349 [0, 0.505]	0.0392 [0, 0.435]	0.0504 [0, 0.806]	0.0409 [0, 0.806]
**Sex**				
Female	2078 (64.9%)	2296 (51.8%)	1961 (61.1%)	6335 (58.4%)
Male	1125 (35.1%)	2140 (48.2%)	1247 (38.9%)	4512 (41.6%)
**Ethnics**				
Black/African American	325 (10.1%)	688 (15.5%)	739 (23.0%)	1752 (16.2%)
White/Caucasian	2739 (85.5%)	3520 (79.4%)	2310 (72.0%)	8569 (79.0%)
Other	138 (4.3%)	224 (5.0%)	157 (4.9%)	519 (4.8%)
Missing	1 (0.0%)	4 (0.1%)	2 (0.1%)	7 (0.1%)
**Levels of education**				
Higher	1591 (49.7%)	1969 (44.4%)	1291 (40.2%)	4851 (44.7%)
Lower	1606 (50.1%)	2464 (55.5%)	1910 (59.5%)	5980 (55.1%)
Missing	6 (0.2%)	3 (0.1%)	7 (0.2%)	16 (0.1%)
**Marital status**				
Never	109 (3.4%)	124 (2.8%)	120 (3.7%)	353 (3.3%)
Partnered	2276 (71.1%)	3298 (74.3%)	2173 (67.7%)	7747 (71.4%)
Seperated/Divorced	488 (15.2%)	585 (13.2%)	482 (15.0%)	1555 (14.3%)
Widow	329 (10.3%)	424 (9.6%)	428 (13.3%)	1181 (10.9%)
Missing	1 (0.0%)	5 (0.1%)	5 (0.2%)	11 (0.1%)
**Year at aged 65**				
1990–2000	1226 (38.3%)	1605 (36.2%)	909 (28.3%)	3740 (34.5%)
2001–2010	1462 (45.6%)	2013 (45.4%)	1495 (46.6%)	4970 (45.8%)
2011–2019	515 (16.1%)	818 (18.4%)	804 (25.1%)	2137 (19.7%)
**Ever drinking alcohol**				
No	1327 (41.4%)	2005 (45.2%)	1787 (55.7%)	5119 (47.2%)
Yes	1876 (58.6%)	2430 (54.8%)	1421 (44.3%)	5727 (52.8%)
Missing	0 (0%)	1 (0.0%)	0 (0%)	1 (0.0%)
**Smoking status**				
Current	717 (22.4%)	717 (16.2%)	347 (10.8%)	1781 (16.4%)
Ever	1209 (37.7%)	1996 (45.0%)	1441 (44.9%)	4646 (42.8%)
Never	1268 (39.6%)	1704 (38.4%)	1408 (43.9%)	4380 (40.4%)
Missing	9 (0.3%)	19 (0.4%)	12 (0.4%)	40 (0.4%)
**Longstanding illness status**				
No	917 (28.6%)	835 (18.8%)	279 (8.7%)	2031 (18.7%)
Yes	2284 (71.3%)	3600 (81.2%)	2928 (91.3%)	8812 (81.2%)
Missing	2 (0.1%)	1 (0.0%)	1 (0.0%)	4 (0.0%)

*BMI* Body mass index

### Association of BMI trajectories and dementia risk

According to the logistic regression model with restricted cubic spline analysis, we found a nonlinear association between the BMI trend in late-middle age and dementia in older age (*P* for no-linear test = 0.038, Fig. S3A) while for the BMI variation in late-middle age, this association was linear (*P* for no-linear test = 0.589, Fig. S3B). As shown in Table 2, compared with participants with ascending BMI trend, a declining BMI trend was significantly associated with increased dementia risk (RR = 1.27, 95% CI = 1.11–1.46, *P* = 0.001). In additional analysis, we found that, in comparison to individuals with stable BMI, an ascending BMI trend is no longer significantly associated with an increased risk of dementia (RR = 0.96, 95% CI = 0.79–1.17, *P* = 0.708). Conversely, a declining BMI trend continues to exhibit a significant association (RR = 1.2, 95% CI = 1–1.44, *P* = 0.048, Table S3), suggesting that while the risk of dementia may be influenced by complex non-linear patterns in BMI during late-middle age, a consistent decline in BMI remains consistently linked to an elevated risk of dementia in older age.

**Table 2:** The association between BMI trend and variation in late-middle age and risk of dementia in older age

Variables	Types	Model 1				Model 2				Model 3				Model 4			
		**No.participants**	**No.event**	**RR (95% CI)**	***P* value**	**No.participants**	**No.event**	**ARR (95% CI)**	***P* value**	**No.participants**	**No.event**	**ARR (95% CI)**	***P* value**	**No.participants**	**No.event**	**ARR (95% CI)**	***P* value**
**BMI trend**	**Ascend**	**6693**	**489**	**Reference**	** < 0.001**	**6672**	**486**	**Reference**	**0.002**	**6643**	**482**	**Reference**	**0.002**	**6643**	**482**	**Reference**	**0.001**
	**Decline**	**4154**	**411**	**1.35 (1.19–1.54)**		**4141**	**409**	**1.23 (1.08–1.4)**		**4125**	**407**	**1.24 (1.08–1.42)**		**4125**	**407**	**1.27 (1.11–1.46)**	
**BMI variation**	**Low**	**6622**	**490**	**Reference**	** < 0.001**	**6601**	**486**	**Reference**	** < 0.001**	**6576**	**481**	**Reference**	** < 0.001**	**6576**	**481**	**Reference**	** < 0.001**
	**High**	**4225**	**410**	**1.31 (1.15–1.5)**		**4212**	**409**	**1.39 (1.22–1.6)**		**4192**	**408**	**1.37 (1.19–1.56)**		**4192**	**408**	**1.36 (1.19–1.56)**	

*BMI* Body mass index

Model 1 was a univariate model for the BMI trend

Model 2 was additionally adjusted for sex, education level, ethnicity, year of aged 65, and marital status

Model 3 was additionally adjusted for smoking status, alcohol consumption, and longstanding illness status

Model 4 was additionally adjusted for the mean BMI group

For participants with high BMI variation, their future dementia risk increased compared with those with low variation BMI (RR = 1.36, 95% CI = 1.19–1.56, *P* < 0.001). For BMI trajectories, we found participants with a declining BMI trend with high variation in the late-middle age had the highest subsequent dementia risk in older age compared with those with an ascending BMI trend with low variation (RR = 1.76, 95% CI = 1.45–2.13, *P* < 0.001, Table 3). All of the associations were independent of the mean BMI in the late-middle age.

**Table 3:** The association between BMI trajectories in late-middle age and risk of dementia in older age

Trajectory	Model1				Model2				Model3				Model 4			
	**No.participants**	**No.event**	**RR (95% CI)**	***P* value**	**No.participants**	**No.event**	**ARR (95% CI)**	***P* value**	**No.participants**	**No.event**	**ARR (95% CI)**	***P* value**	**No.participants**	**No.event**	**ARR (95% CI)**	***P* value**
**Ascend BMI with low variation**	**3932**	**264**	**Reference**		**3920**	**262**	**Reference**		**3902**	**258**	**Reference**		**3902**	**258**	**Reference**	
**Decline BMI with low variation**	**2690**	**226**	**1.25 (1.05–1.49)**	**0.013**	**2681**	**224**	**1.13 (0.95–1.36)**	**0.17**	**2674**	**223**	**1.16 (0.97–1.39)**	**0.113**	**2674**	**223**	**1.18 (0.98–1.42)**	**0.076**
**Ascend BMI with high variation**	**2761**	**225**	**1.21 (1.02–1.45)**	**0.033**	**2752**	**224**	**1.28 (1.07–1.54)**	**0.007**	**2741**	**224**	**1.27 (1.06–1.53)**	**0.01**	**2741**	**224**	**1.27 (1.06–1.52)**	**0.011**
**Decline BMI with high variation**	**1464**	**185**	**1.88 (1.56–2.27)**	** < 0.001**	**1460**	**185**	**1.79 (1.48–2.16)**	** < 0.001**	**1451**	**184**	**1.75 (1.45–2.12)**	** < 0.001**	**1451**	**184**	**1.76 (1.45–2.13)**	** < 0.001**

*BMI* Body mass index

Model 1 was a univariate model for the BMI trajectory

Model 2 was additionally adjusted for sex, education, ethnicity, year of aged 65, and marital status

Model 3 was additionally adjusted for smoking status, alcohol consumption, and longstanding illness status. Model 4 was additionally adjusted for the mean BMI group

**Fig. 1:**
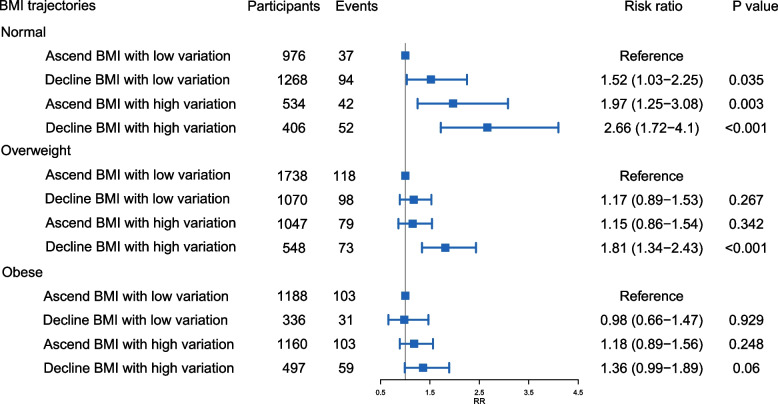
The association between BMI trajectories and risk of dementia stratified by mean BMI groups. Models were adjusted for sex, ethnicity, levels of education, year of age 65, marital status, smoking status, alcohol consumption status, history of medical conditions, and mean BMI group

In addition, we conducted stratified analyses on BMI trajectories and dementia risk based on each individual's mean BMI (Fig. 1). The strongest association between a declining BMI trend with high variation and elevated dementia risk was observed in normal BMI group (RR = 2.66, 95% CI = 1.72–4.1, *P* < 0.001) while attenuated associations of a declining BMI trend with high variation and elevated dementia risk were observed in obese BMI group (RR = 1.36, 95% CI = 0.99–1.89, *P* = 0.06).

### Secondary analyses on the subcohort with PGS

**Fig. 2:**
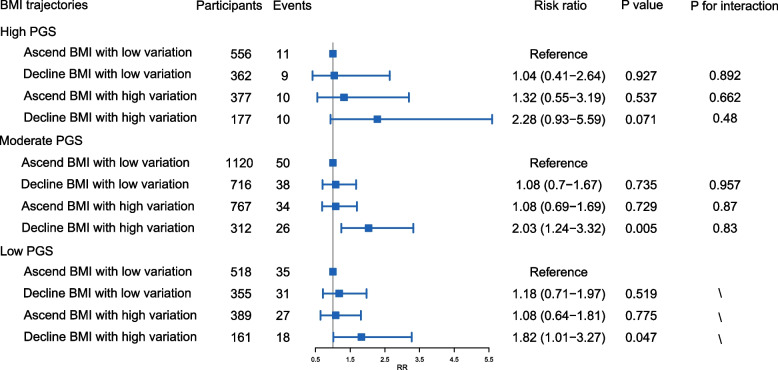
The association between BMI trajectories and dementia risk stratified by PGS. Models were adjusted for sex, levels of education, year of age 65, marital status, smoking status, alcohol consumption, history of medical conditions, mean BMI group, and PC1-5. PGS: polygenetic score

A total of 5,845 participants of European ancestry were included in the subcohort analysis involving the PGS. Detailed participant characteristics are provided in Table S4. High PGS scores for general cognitive function were significantly associated with a reduced risk of dementia in older age (RR = 0.37, 95% CI = 0.25–0.53, *P* < 0.001, Table S5), indicating the PGS for general cognitive function's predictive value for subsequent dementia risk in older individuals. Adjusted for PGS and PC1-5, a decline in BMI with high variation was significantly linked to an increased dementia risk in older age (ARR = 2.02, 95% CI = 1.44–2.85, *P* < 0.001, Figure S4), implying an independent association, not influenced by participants' genetic predisposition to general cognitive function. Similar associations were observed when stratifying the cohort based on their PGS (Fig. 2), suggesting consistency in the relationship between BMI trajectories and subsequent dementia risk across different genetic predisposition groups.

### Subgroup and sensitivity analysis

Subgroup analyses were conducted to assess the potential interactions of BMI variation and trajectories in late-middle age. Modified effects on education level was found in the association between BMI trajectories and dementia in older age (*P* for interaction < 0.001, Figure S5). The results showed that participants with higher levels of education were more likely to show an enhanced association between BMI trajectories and dementia risk. The results of sensitivity analyses were consistent with the main analyses (Table S6).

## Discussion

In the present study, we comprehensively assessed the association between BMI trajectories in late-middle age and subsequent dementia risk in older age in a population-based cohort with 26 years of follow-up. A declining BMI trend with high variation in late-middle age was significantly associated with increased subsequent dementia risk in older age, especially in participants in the normal-BMI group. Genetic performance regarding cognition function did not interact with BMI trajectories on the risk of dementia in older age.

Several studies have focused on the association of BMI changes with dementia risk [28, 29]. Reverse causation may exist between BMI changes and dementia risk because weight loss might be a preclinical characteristic of dementia and may occur at least ten years before the diagnosis of the disease [30–32]. Russ et al. classified 33,083 participants from The Harvard Alumni Health Study population into early decliners whose BMIs dropped around the age of 50 years and late decliners whose BMIs dropped 20 years later using latent class mixed models [33]. They found that those who experienced an early decline in BMI were more likely to have dementia-related death [33]. However, because these results were reported from highly selective samples with higher educational attainment and socioeconomic status, further studies are warranted. Moreover, the results from the Mayo Clinic Study of Aging suggested an increased rate of weight loss from middle age to older age was associated with incident mild cognitive impairment [34]. Similar results were found by Wagner et al. [29] and Fitzpatrick et al. [28], which shows BMI trajectory in midlife and late-life or before dementia were associated with subsequent dementia risks. The results of the BMI trend from our study were firstly revealed that decline BMI trend in late-middle age was associated with increased subsequent dementia risk in older age regardless of the mean BMI or the inherent genetic risk.

Studies have revealed that BMI changes have a reverse J-shaped association with cognitive impairment and dementia [17, 35]. A recent study also demonstrated that in participants with new-onset diabetes, not only weight loss, but also weight gain was associated with an increased risk of all-cause dementia [36]. Li et al. found that declining BMI patterns in mid- to late life were linked to a higher risk of dementia, particularly among individuals who experienced an initial increase in BMI during their early middle age [37]. Our study steps forward, assuming that BMI variation itself, which not only considers relatively increased or decreased BMI values, but also BMI fluctuation, might be associated with dementia. Such associations have been previously found by Lan et al., who showed that substantial body weight fluctuation during late-life was associated with cognitive decline [38]. Our results showed that high BMI variation in late-middle age was significantly associated with increased subsequent dementia risk in older age. Based on BMI trend and variation results, we constructed BMI trajectories to help us further identify the individuals at high risk of dementia using their BMI information in late-middle age. A declining BMI trend with high variation was strongly associated with dementia risk in older age. The robustness of this association was tested, and the results were consistent between sensitivity analyses and subgroup analyses.

The PGS for general cognitive function represents the genetic contribution of cognitive function and it could explain up to 4.3% of the variance in general cognitive function [26]. Genetic overlap was discovered between general cognitive function and health variables, including hypertension and longevity [26]. Previous studies have shown that the genetic risk of dementia has no modification effects on the association of healthy lifestyle, cardiovascular health, and sleep duration with dementia incidence [39–41]. In the present study, we also observed that the association of BMI trajectories and dementia risk was independent of the PGS for general cognitive function, without any modification effects.

The present study has some strengths. First, by using the HRS, which involved a nationally representative longitudinal cohort with repeated measurements and long-term follow-up, we could investigate dynamic BMI changes in late-middle life and subsequent dementia risk in older age with sufficient statistical power and good generalizability in the United States. Second, BMI trends were quantified by using a multilevel model, which not only allowed us to make maximum use of BMI information for each individual in late-middle age but also considered both the BMI trend of the general cohort and each individual at the same time [42]. Furthermore, our study took the inherited genetic status of each participant into consideration, which largely improved the interpretability of the models.

It is important to underscore the potential impact of missing data on our dementia risk assessment, particularly within the context of BMI trajectories. In our study, we evaluated dementia risk based on participants' cognitive test results, and this assessment may be influenced by individuals who did not provide follow-up cognitive information. This introduces a potential source of bias, as the outcome of dementia risk becomes uncertain when cognitive assessments are unavailable. Moreover, the absence of data may also be attributed to unmeasured health-related events, such as cardiovascular incidents associated with ascending BMI trajectories. Given that dementia typically manifests at older ages compared to many other health conditions, it is essential to pay attention to and conduct further studies on the complexities of missing data in this context.

It should be noticed the impact of missing data on dementia risk assessment, particularly in the context of BMI trajectories. In our study we assessed dementia according to cognitive test of participants itself and its assessment may be influenced by participants who did not provide cognitive follow-up information. This introduces a potential source of bias, as the outcome of dementia risk becomes uncertain when cognitive assessments are unavailable. Also, unmeasured health-related events, such as cardiovascular events associated with ascending BMI trajectories, may have contributed to missing data, given dementia is indeed expected to manifest at older ages than many other diseases. To partially address this issue, we employed multiple imputation to validate our findings through sensitivity analyses. The results of these analyses revealed that, after multiple imputation, the significance and estimate remained largely consistent with the main analyses, suggesting the robustness of our study (Sensitivity analysis 4, Figure S6).

Several other limitations warrant consideration. Based on the nature of the cohort study, unsurveyed covariates and potential residuals may exist and confounded the association between BMI trajectories and dementia. Additionally, the BMI records were self-reported by the participants, and their smoking and drinking histories were acquired without an exact consumption amount, which may lead to recall bias. Furthermore, it is essential to acknowledge that our model may not be capable of capturing potential bidirectional BMI changes over time, as individuals in such cases may have estimated slopes that are close to zero. For these participants, our model may not accurately represent the true underlying patter. Last but not least, the assessment of dementia was dependent on the cognitive test conducted at every wave, which may introduce bias in the Cox models in our sensitivity analysis.

## Conclusion

In general, the present study revealed a declining BMI trend with high variation was significantly associated with dementia risk in older age. The genetic performance regarding general cognitive function was independent of this association with no potential modifying effects. The results suggest that BMI trajectories in late-middle age could be used as a marker to identify individuals with a high risk of dementia.

### Supplementary Information

**Additional file 1: Figure S1.** The flow chart of the study population. **Figure S2.** The correlation between BMI trend, BMI variations and average BMIs. Spearman's rank order correlation was utilized to assess these relationships. The numbers represent the Spearman's rank coefficient, with "***" indicating statistical significance at *p* < 0.001. **Figure S3.** The restricted cubic splines showing the non-linear association test between the BMI trend (A) and BMI variation (B) in late-middle age and dementia in older age. **Figure S4.** The association between BMI trajectories in late-middle age and risk of dementia in older age in genetic sub-cohort analyses after adjusted for the polygenetic score of cognition performance. **Figure S5.** The association between BMI trajectories in late-middle age and risk of dementia in older age stratified by pre-defined subgroups. **Table S1.** Comparison of covariates between participants excluded due to lack of followup cognitive information and included study participants. **Table S2.** Baseline characteristics of BMI indicators, stratified by the cognitive status after age 65. **Table S3.** The association between BMI trend (three-category) in late-middle age and risk of dementia in older age. **Table S4.** The baseline characteristics of participants included in the genetic analyses. **Table S5.** The association between polygenetic score (PGS) and risk of dementia in older age. **Table S6.** Sensitivity analyses BMI trajectories in late-middle age and risk of dementia in older age.

## Data Availability

The HRS database is publicly available and can be accessed by submitting a reasonable request. The model-related data supporting the findings of this study are available within the article and its supplementary materials.

## Abbreviations

BMI: Body mass index
HRS: Health and Retirement Study
NIA: National Institute on Aging
ADAMS: Aging, Demographics, and Memory Study
PGS: Polygenic score
PC: Principal component
ARRs: Adjusted risk ratios
CIs: Confidence intervals
