# Supplementary material for: Association between BMI trajectories in late-middle age and subsequent dementia risk in older age: a 26-year population-based cohort study
Source: BMC Geriatr. 2023 Nov 24;23:773. doi: 10.1186/s12877-023-04483-z (PMC10675868; doi:10.1186/s12877-023-04483-z)
Supplement: Supplementary file 1 — Additional file 1: Figure S1. The flow chart of the study population. Figure S2. The correlation between BMI trend, BMI variations and average BMIs. Spearman's rank order correlation was utilized to assess these relationships. The numbers represent the Spearman's rank coefficient, with "***" indicating statistical significance at p < 0.001. Figure S3. The restricted cubic splines showing the non-linear association test between the BMI trend (A) and BMI variation (B) in late-middle age and dementia in older age. Figure S4. The association between BMI trajectories in late-middle age and risk of dementia in older age in genetic sub-cohort analyses after adjusted for the polygenetic score of cognition performance. Figure S5. The association between BMI trajectories in late-middle age and risk of dementia in older age stratified by pre-defined subgroups. Table S1. Comparison of covariates between participants excluded due to lack of followup cognitive information and included study participants. Table S2. Baseline characteristics of BMI indicators, stratified by the cognitive status after age 65. Table S3. The association between BMI trend (three-category) in late-middle age and risk of dementia in older age. Table S4. The baseline characteristics of participants included in the genetic analyses. Table S5. The association between polygenetic score (PGS) and risk of dementia in older age. Table S6. Sensitivity analyses BMI trajectories in late-middle age and risk of dementia in older age. [file 12877_2023_4483_MOESM1_ESM.pdf]

Supplementary material

Figure.S1 The flow chart of the study population.

Figure.S2 The correlation between BMI trend, BMI variations and average BMIs. Spearman's rank order correlation was utilized to assess these relationships. The numbers represent the Spearman's rank coefficient, with "*" indicating statistical significance at p < 0.001.**

Figure.S3 The restricted cubic splines showing the non-linear association test between the BMI trend (A) and BMI variation (B) in late-middle age and dementia in older age.

Figure.S4 The association between BMI trajectories in late-middle age and risk of dementia in older age in genetic sub-cohort analyses after adjusted for the polygenetic score of cognition performance.

Figure.S5 The association between BMI trajectories in late-middle age and risk of dementia in older age stratified by pre-defined subgroups.

Table.S1 Comparison of covariates between participants excluded due to lack of followup cognitive information and included study participants.

Table S2. Baseline characteristics of BMI indicators, stratified by the cognitive status after age 65.

Table S3. The association between BMI trend (three-category) in late-middle age and risk of dementia in older age.

Table S4. The baseline characteristics of participants included in the genetic analyses.

Table S5. The association between polygenetic score (PGS) and risk of dementia in older age.

Figure.S1 The flow chart of the study population.

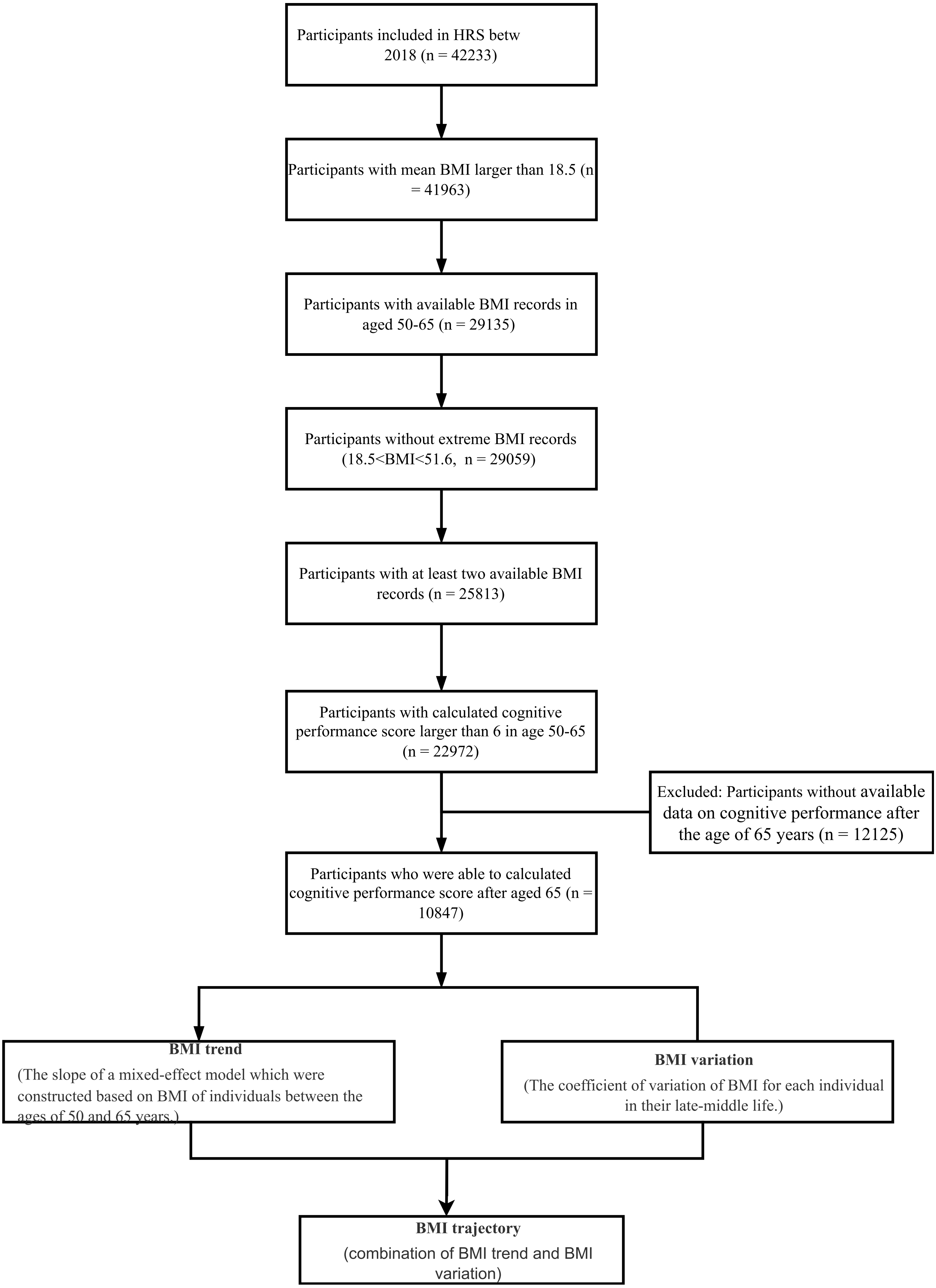

Figure S2 The correlation between BMI trend, BMI variations and average BMIs. Spearman's rank-order correlation was utilized to assess these relationships. The numbers represent the Spearman's rank coefficient, with "***" indicating statistical significance at $p < 0.001$.

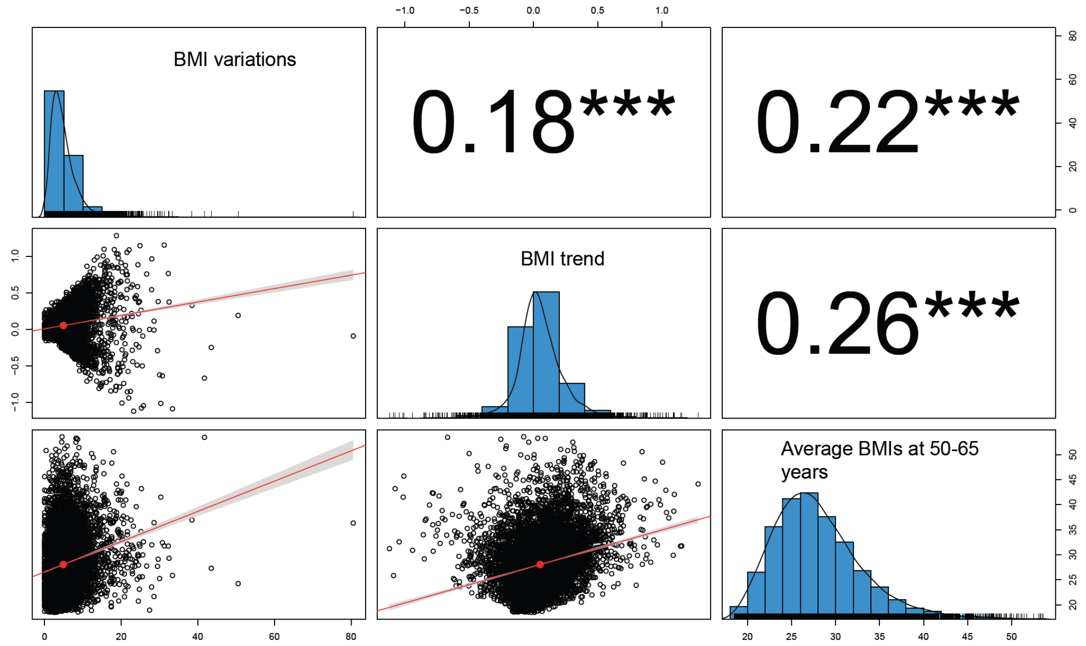

Figure.S3 The restricted cubic splines showing the non-linear association test between the BMI trend (A) and BMI variation (B) in late-middle age and dementia in older age.

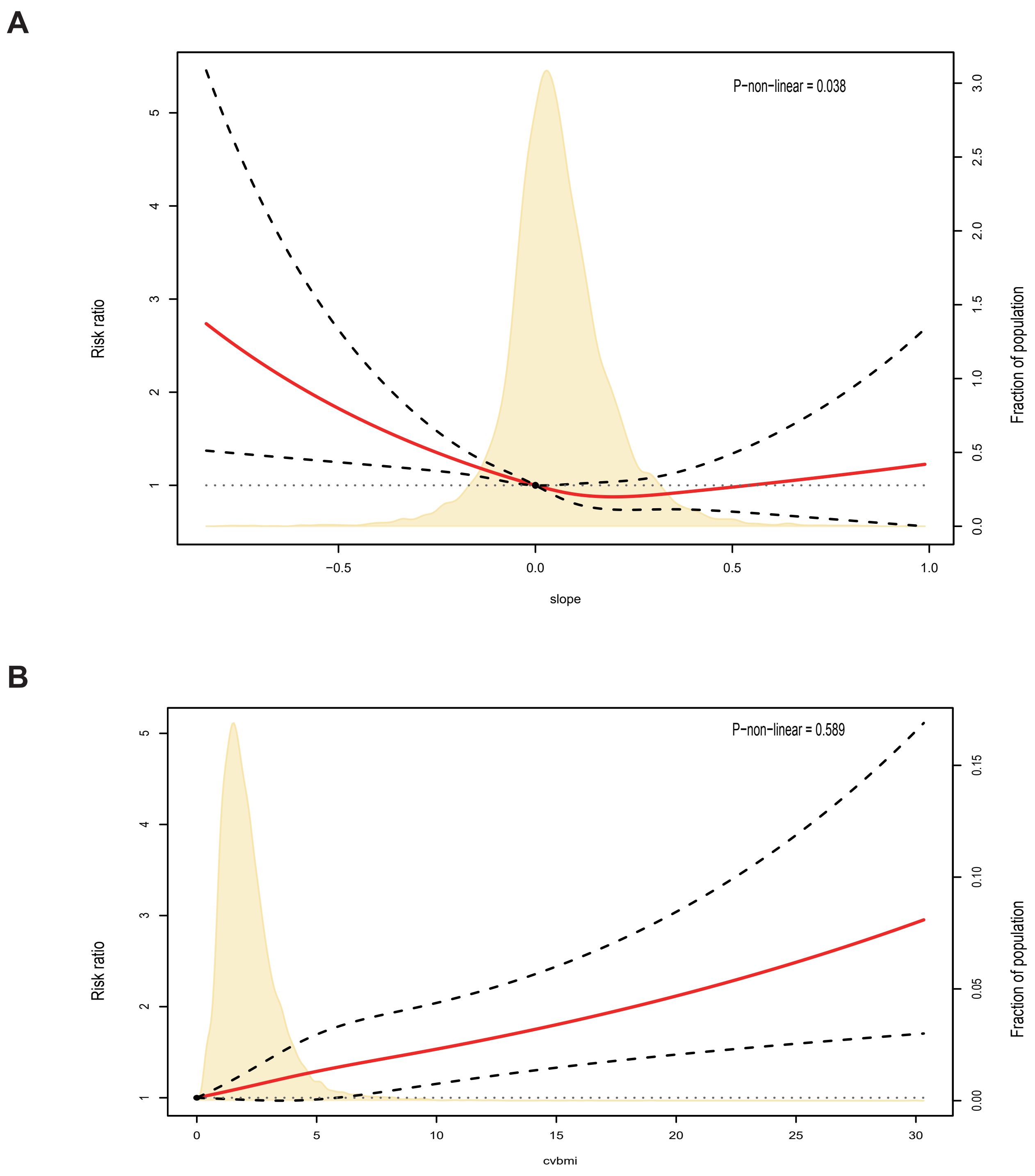

Figure.S4 The association between BMI trajectories in late-middle age and risk of dementia in older age in genetic sub-cohort analyses after adjusted for the polygenetic score of cognition performance.

BMI trajectories	Participants	Events	Risk ratio	P value
Ascend BMI with low variation	2194	96	Reference	
Decline BMI with low variation	1433	78	1.07 (0.79−1.46)	0.657
Ascend BMI with high variation	1533	71	1.11 (0.81−1.53)	0.501
Decline BMI with high variation	650	54	2.02 (1.44−2.85)	<0.001

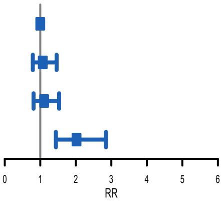

Figure.S5 The association between BMI trajectories in late-middle age and risk of dementia in older age stratified by pre-defined subgroups.

Subgroups	Participants	Events	Risk ratio	P value	P for interaction
Sex					0.339
Female	6231	537			
Ascend BMI with low variation			Reference		
Decline BMI with low variation			1.29(1−1.66)	0.049	
Ascend BMI with high variation			1.31(1.04−1.66)	0.024	
Decline BMI with high variation			1.86(1.46−2.37)	<0.001	
Male	4449	344			
Ascend BMI with low variation			Reference		
Decline BMI with low variation			1.08(0.82−1.43)	0.564	
Ascend BMI with high variation			1.1(0.82−1.48)	0.535	
Decline BMI with high variation			1.19(0.86−1.67)	0.295	
Ehnicity					0.998
White/Caucasian	8449	523			
Ascend BMI with low variation			Reference		
Decline BMI with low variation			1.2(0.94−1.53)	0.142	
Ascend BMI with high variation			1.21(0.96−1.54)	0.113	
Decline BMI with high variation			1.84(1.44−2.36)	<0.001	
Other	2231	358			
Ascend BMI with low variation			Reference		
Decline BMI with low variation			1.24(0.93−1.66)	0.139	
Ascend BMI with high variation			1.23(0.92−1.63)	0.155	
Decline BMI with high variation			1.31(0.96−1.78)	0.091	
Levels of education					<0.001
Higher	4789	154			
Ascend BMI with low variation			Reference		
Decline BMI with low variation			2.15(1.36−3.4)	0.001	
Ascend BMI with high variation			1.93(1.17−3.16)	0.01	
Decline BMI with high variation			3.32(2.04−5.4)	<0.001	
Lower	5891	727			
Ascend BMI with low variation			Reference		
Decline BMI with low variation			1.06(0.86−1.3)	0.597	
Ascend BMI with high variation			1.19(0.98−1.45)	0.078	
Decline BMI with high variation			1.59(1.28−1.96)	<0.001	
Smoking status					0.353
Ever	6349	552			
Ascend BMI with low variation			Reference		
Decline BMI with low variation			1.13(0.89−1.43)	0.311	
Ascend BMI with high variation			1.09(0.86−1.38)	0.465	
Decline BMI with high variation			1.58(1.24−2.01)	<0.001	
Never	4331	329			
Ascend BMI with low variation			Reference		
Decline BMI with low variation			1.35(0.99−1.82)	0.054	
Ascend BMI with high variation			1.48(1.1−1.99)	0.01	
Decline BMI with high variation			1.56(1.13−2.16)	0.007	
Ever drinking alcohol					0.204
No	5027	561			
Ascend BMI with low variation			Reference		
Decline BMI with low variation			1.12(0.88−1.42)	0.346	
Ascend BMI with high variation			1.06(0.84−1.32)	0.64	
Decline BMI with high variation			1.47(1.16−1.85)	0.001	
Yes	5653	320			
Ascend BMI with low variation			Reference		
Decline BMI with low variation			1.34(0.99−1.82)	0.057	
Ascend BMI with high variation			1.55(1.14−2.12)	0.005	
Decline BMI with high variation			1.81(1.28−2.56)	<0.001	
Marital status					0.852
Partnered	7641	550			
Ascend BMI with low variation			Reference		
Decline BMI with low variation			1.19(0.95−1.49)	0.128	
Ascend BMI with high variation			1.07(0.85−1.35)	0.567	
Decline BMI with high variation			1.3(1−1.69)	0.046	
Other	3039	331			
Ascend BMI with low variation			Reference		
Decline BMI with low variation			1.26(0.9−1.76)	0.187	
Ascend BMI with high variation			1.52(1.12−2.07)	0.007	
Decline BMI with high variation			2.12(1.56−2.88)	<0.001	

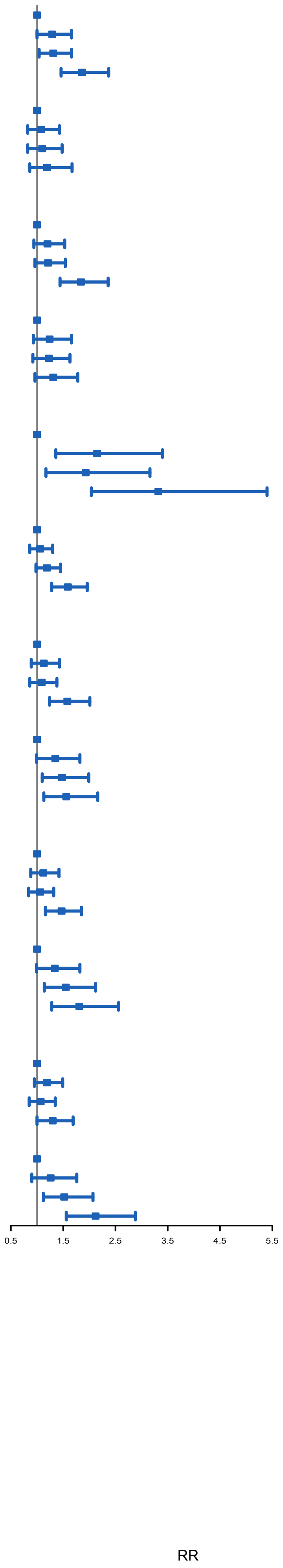

Table S1 Comparison of covariates between participants excluded due to lack of follow-up cognitive information and included study participants.

	Excluded participants	Included participants	Overall
	(N=12125)	(N=10847)	(N=22972)
**Sex**			
Female	6668 (55.0%)	6335 (58.4%)	13003 (56.6%)
Male	5457 (45.0%)	4512 (41.6%)	9969 (43.4%)
**Ethnics**			
Black/African American	2919 (24.1%)	1752 (16.2%)	4671 (20.3%)
White/Caucasian	1655 (13.6%)	519 (4.8%)	2174 (9.5%)
Other	7497 (61.8%)	8569 (79.0%)	16066 (69.9%)
Missing	54 (0.4%)	7 (0.1%)	61 (0.3%)
**Levels of education**			
Higher	6483 (53.5%)	4851 (44.7%)	11334 (49.3%)
Lower	5571 (45.9%)	5980 (55.1%)	11551 (50.3%)
Missing	71 (0.6%)	16 (0.1%)	87 (0.4%)
**Marital status**			
Never	986 (8.1%)	353 (3.3%)	1339 (5.8%)
Partnered	8033 (66.3%)	7747 (71.4%)	15780 (68.7%)
Seperated/Divorced	2333 (19.2%)	1555 (14.3%)	3888 (16.9%)
Widow	749 (6.2%)	1181 (10.9%)	1930 (8.4%)
Missing	24 (0.2%)	11 (0.1%)	35 (0.2%)
**Year at aged 65**			
1990-2000	1085 (8.9%)	3740 (34.5%)	4825 (21.0%)
2001-2010	1057 (8.7%)	4970 (45.8%)	6027 (26.2%)
2011-2019	9983 (82.3%)	2137 (19.7%)	12120 (52.8%)
**Ever drinking alcohol**			
No	4873 (40.2%)	5119 (47.2%)	9992 (43.5%)
Yes	7242 (59.7%)	5727 (52.8%)	12969 (56.5%)
Missing	10 (0.1%)	1 (0.0%)	11 (0.0%)
**Smoking status**			
Current	2417 (19.9%)	1781 (16.4%)	4198 (18.3%)
Ever	4474 (36.9%)	4646 (42.8%)	9120 (39.7%)
Never	5210 (43.0%)	4380 (40.4%)	9590 (41.7%)
Missing	24 (0.2%)	40 (0.4%)	64 (0.3%)
**History of medical conditions**			
No	2761 (22.8%)	2031 (18.7%)	4792 (20.9%)
Yes	9362 (77.2%)	8812 (81.2%)	18174 (79.1%)
Missing	2 (0.0%)	4 (0.0%)	6 (0.0%)
**Mean BMI group**			
Normal	2850 (23.5%)	3203 (29.5%)	6053 (26.3%)

Overweight	4489 (37.0%)	4436 (40.9%)	8925 (38.9%)
Obese	4786 (39.5%)	3208 (29.6%)	7994 (34.8%)

Table S2. Baseline characteristics of BMI indicators, stratified by the cognitive status after age 65.

	**Non-dementia (N=9947)**	**Dementia (N=900)**	**Overall (N=10847)**
**BMI trend at age 50-65**			
Decline	3743 (37.6%)	411 (45.7%)	4154 (38.3%)
Ascend	6204 (62.4%)	489 (54.3%)	6693 (61.7%)
**BMI variation at age 50-65**			
Mean (SD)	4.85 (3.61)	5.33 (4.00)	4.89 (3.65)
Median [Min, Max]	4.05 [0, 80.6]	4.50 [0, 28.0]	4.09 [0, 80.6]
**Mean BMI at age 50-65**			
Normal	2974 (29.9%)	229 (25.4%)	3203 (29.5%)
Obese	2908 (29.2%)	300 (33.3%)	3208 (29.6%)
Overweight	4065 (40.9%)	371 (41.2%)	4436 (40.9%)

Table S3. The association between BMI trend (three-category) in late-middle age and risk of dementia in older age.

Variables	Types	Model1				Model2				Model3				Model 4			
		**No.participants**	**No.event**	**RR (95% CI)**	**P value**	**No.participants**	**No.event**	**ARR (95% CI)**	**P value**	**No.participants**	**No.event**	**ARR (95% CI)**	**P value**	**No.participants**	**No.event**	**ARR (95% CI)**	**P value**
	**Stable**	2169	164	Reference		2162	163	Reference		2151	163	Reference		2151	163	Reference	
	**Ascend**	4339	421	0.96 (0.8-1.16)	0.673	4325	313	1.02 (0.84-1.23)	0.856	4308	310	0.99 (0.82-1.2)	0.92	4308	310	0.96 (0.79-1.17)	0.708
**BMI trend**	**Decline**	4339	315	1.28 (1.07-1.54)	0.007	4326	419	1.2 (1-1.44)	0.044	4309	416	1.19 (0.99-1.43)	0.06	4309	416	1.2 (1-1.44)	0.048

Table S4. The baseline characteristics of participants included in the genetic analyses.

	Normal (N=1901)	Overweight (N=2393)	Obese (N=1551)	Overall (N=5845)
**BMI trend**				
Decline	921 (48.4%)	795 (33.2%)	381 (24.6%)	2097 (35.9%)
Ascend	980 (51.6%)	1598 (66.8%)	1170 (75.4%)	3748 (64.1%)
**BMI variation**				
Mean (SD)	4.02 (2.84)	4.61 (3.15)	5.76 (3.62)	4.72 (3.26)
Median [Min, Max]	3.43 [0, 28.5]	3.90 [0, 29.6]	5.08 [0, 31.2]	4.02 [0, 31.2]
**PGS of general cognition function**				
Low	470 (24.7%)	567 (23.7%)	391 (25.2%)	1428 (24.4%)
Moderate	927 (48.8%)	1220 (51.0%)	789 (50.9%)	2936 (50.2%)
High	504 (26.5%)	606 (25.3%)	371 (23.9%)	1481 (25.3%)
**Sex**				
Female	1279 (67.3%)	1204 (50.3%)	883 (56.9%)	3366 (57.6%)
Male	622 (32.7%)	1189 (49.7%)	668 (43.1%)	2479 (42.4%)
**Levels of education**				
Higher	1033 (54.3%)	1186 (49.6%)	709 (45.7%)	2928 (50.1%)
Lower	866 (45.6%)	1205 (50.4%)	837 (54.0%)	2908 (49.8%)
Missing	2 (0.1%)	2 (0.1%)	5 (0.3%)	9 (0.2%)
**Marital status**				
Never	46 (2.4%)	47 (2.0%)	40 (2.6%)	133 (2.3%)
Partnered	1454 (76.5%)	1926 (80.5%)	1170 (75.4%)	4550 (77.8%)
Seperated/Divorced	230 (12.1%)	233 (9.7%)	174 (11.2%)	637 (10.9%)
Widow	171 (9.0%)	187 (7.8%)	164 (10.6%)	522 (8.9%)
Missing	0 (0%)	0 (0%)	3 (0.2%)	3 (0.1%)
**Year at aged 65**				
1990-2000	682 (35.9%)	793 (33.1%)	399 (25.7%)	1874 (32.1%)
2001-2010	946 (49.8%)	1180 (49.3%)	794 (51.2%)	2920 (50.0%)
2011-2019	273 (14.4%)	420 (17.6%)	358 (23.1%)	1051 (18.0%)
**Ever drinking alcohol**				
No	701 (36.9%)	943 (39.4%)	766 (49.4%)	2410 (41.2%)
Yes	1200 (63.1%)	1450 (60.6%)	785 (50.6%)	3435 (58.8%)
**Smoking status**				
Current	360 (18.9%)	335 (14.0%)	158 (10.2%)	853 (14.6%)
Ever	744 (39.1%)	1110 (46.4%)	725 (46.7%)	2579 (44.1%)
Never	791 (41.6%)	938 (39.2%)	664 (42.8%)	2393 (40.9%)
Missing	6 (0.3%)	10 (0.4%)	4 (0.3%)	20 (0.3%)
**Longstanding illness status**				
No	565 (29.7%)	480 (20.1%)	130 (8.4%)	1175 (20.1%)
Yes	1334 (70.2%)	1912 (79.9%)	1421 (91.6%)	4667 (79.8%)
Missing	2 (0.1%)	1 (0.0%)	0 (0%)	3 (0.1%)

PGS: polygenetic score, BMI: Body mass index

Table S5. The association between polygenetic score (PGS) and risk of dementia in older age.

PGS for cognition performance	Model1				Model2				Model3				Model 4			
	No.participants	No.event	RR (95% CI)	P value	No.participants	No.event	ARR (95% CI)	P value	No.participants	No.event	ARR (95% CI)	P value	No.participants	No.event	ARR (95% CI)	P value
Low	1428	112	Reference		1425	112	Reference		1423	111	Reference		1423	111	Reference	
Moderate	2936	151	0.62 (0.49-0.8)	<0.001	2928	151	0.68 (0.53-0.88)	0.003	2915	148	0.68 (0.53-0.88)	0.003	2915	148	0.68 (0.53-0.88)	0.003
High	1481	41	0.31 (0.22-0.45)	<0.001	1480	41	0.36 (0.25-0.53)	<0.001	1472	40	0.36 (0.25-0.53)	<0.001	1472	40	0.37 (0.25-0.53)	<0.001

Model 1 was adjusted for PC1-5. Model 2 was additionally adjusted for sex, education, year of enrollment, marital status. Model 3 was additionally adjusted for smoking status, alcohol consumption, and longstanding illness status. Model 4 was additionally adjusted for the mean BMI group. PGS: polygenetic score, BMI: Body mass index

Table S6. Sensitivity analyses BMI trajectories in late-middle age and risk of dementia in older age.

Trajectories	Sensitivity analysis 1		Sensitivity analysis 2		Sensitivity analysis 3		Sensitivity analysis 4	
	ARR (95% CI)	P value	ARR (95% CI)	P value	HR (95% CI)	P value	ARR (95% CI)	P value
Ascend BMI with low variation	Reference		Reference		Reference		Reference	
Decline BMI with low variation	1.18 (0.98-1.42)	0.076	0.32 (0.08-1.3)	0.111	1.23(1.02-1.48)	0.026	1.24 (1.02-1.51)	0.032
Ascend BMI with high variation	1.27 (1.06-1.52)	0.011	1.26 (1.08-1.47)	0.003	1.46(1.21-1.75)	<0.001	1.33 (1.09-1.63)	0.005
Decline BMI with high variation	1.76 (1.45-2.13)	<0.001	1.59 (1.3-1.95)	<0.001	2.38(1.96-2.88)	<0.001	1.98 (1.6-2.46)	<0.001

Sensitivity analyses 1 included participants who had at least three BMI records in late-middle age. Sensitivity analyses 2 used the exact changes in BMI between the last record and the first record replaced the BMI trend. Sensitivity analyses 3 constructed Cox proportional hazard model to investigate the association between BMI trajectories on dementia. Sensitivity analyses 4 used multiple imputation to validate the estimates obtained from the complete case analyses. BMI: Body mass index
